# Pseudomesotheliomatous Adenocarcinoma Presenting as Hemothorax and Mimicking Mesothelioma: A Case Study

**DOI:** 10.7759/cureus.106699

**Published:** 2026-04-09

**Authors:** Mohamed IJIM, Sarah Keddabi, Chaynez Rachid, Oussama Fikri, Lamyae Amro

**Affiliations:** 1 Pulmonology Department, University Hospital Center Mohammed VI, Arrazi Hospital, Faculty of Medicine and Pharmacy of Marrakech, Morpho Sciences Research Laboratory, Cadi Ayyad University, Marrakech, MAR

**Keywords:** chemotherapy, differential diagnosis, hemothorax, immunohistochemistry, lung adenocarcinoma, pseudomesotheliomatous carcinoma

## Abstract

Pseudomesotheliomatous carcinoma of the lung (PCL) is a rare and aggressive subtype of peripheral lung adenocarcinoma characterized by diffuse pleural involvement, clinically and radiologically mimicking malignant pleural mesothelioma. We report the case of a 53-year-old Moroccan man presenting with hemothorax, a rare initial manifestation of PCL. Radiological imaging revealed nodular pleural thickening with loculated pleural effusion, while histopathological and immunohistochemical analyses confirmed pseudomesotheliomatous lung adenocarcinoma, distinguished by positive epithelial markers (TTF-1, Napsin A) and negative mesothelial markers. Despite initial management with chest drainage and systemic chemotherapy, the prognosis remained poor, consistent with the aggressive nature of this disease. This case highlights the diagnostic challenge posed by PCL due to its clinical and radiological resemblance to mesothelioma and underscores the essential role of immunohistochemistry in establishing an accurate diagnosis. Increased awareness of this entity is critical for appropriate patient management and guiding future therapeutic strategies.

## Introduction

Pseudomesotheliomatous carcinoma of the lung (PCL) [1,2] is a rare and aggressive subtype of peripheral lung adenocarcinoma presenting with diffuse pleural involvement, often mimicking malignant pleural mesothelioma both clinically and radiologically. It typically manifests with pleural thickening and effusion, often without a detectable parenchymal lung mass on imaging, making differential diagnosis particularly challenging. Accurate diagnosis relies on a multidisciplinary approach, combining clinical evaluation (e.g., chest pain, dyspnea, pleural effusion), radiologic imaging, histopathology, and crucially, immunohistochemical profiling to distinguish PCL from true mesothelioma and other pleural malignancies.

The prognosis of PCL is generally poor due to its diffuse spread, rapid progression, and limited response to conventional therapies. While treatment strategies are similar to those used for other non-small cell lung cancers, the pleural-based dissemination often limits curative options. Recent molecular studies aim to identify genetic alterations and biomarkers specific to PCL, which may eventually contribute to more accurate diagnosis and the development of targeted therapies [3-5].

## Case presentation

A 53-year-old Moroccan man, chronic smoker (30 pack-years, ceased 20 days prior), had a known history of chronic dyspnea and bronchitis, with prolonged exposure to wood smoke and poultry/livestock droppings.

Two months prior to admission, he developed atypical left lateral thoracic pain, rated 4/10 on the visual analog scale (VAS), associated with dyspnea classified as stage II according to Sadoul [6]. He also reported a dry cough that became productive 10 days before admission, yielding whitish sputum occasionally streaked with blood. Symptoms evolved in an afebrile context, with general deterioration (non-quantified weight loss, anorexia, and asthenia).

On clinical examination at admission, the patient was conscious, with a WHO performance status of 0, respiratory rate was 26 breaths per minute, heart rate 92 beats per minute, oxygen saturation 94% on room air, and blood pressure 110/70 mmHg. Pulmonary examination revealed left basal pleural fluid effusion.

**Figure 1:**
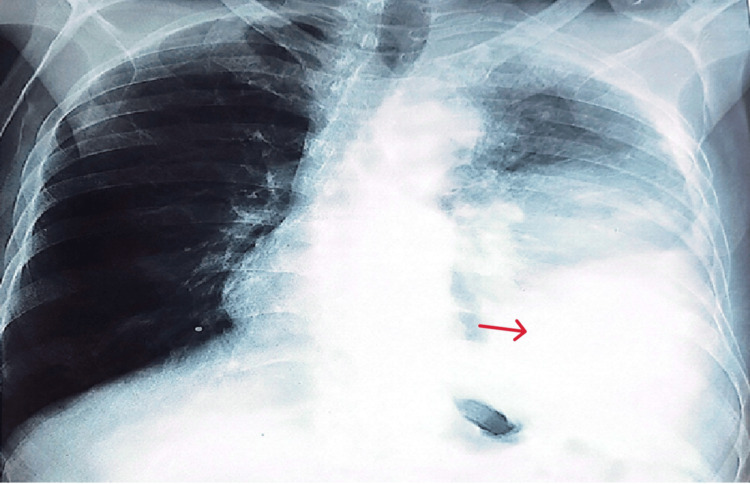
Chest X-ray showing a left basal loculated pleural effusion with an overlying heterogeneous retractile opacity. The red arrow indicates pleural effusion.

A chest X-ray performed at admission revealed a dense, homogeneous opacity of fluid density in the left lower thoracic region, with rounded margins, consistent with loculated pleural effusion, surmounted by a heterogeneous, retractile opacity (Figure 1).

The hemostasis workup was unremarkable (prothrombin time and platelet count). Diagnostic thoracentesis revealed hemorrhagic pleural fluid. Given this macroscopic appearance, simultaneous hematocrit measurements were performed in both pleural fluid and peripheral blood, confirming hemothorax (Table 1). A pleural biopsy and chest tube drainage were subsequently carried out. A 26 French Joly chest tube was inserted in the left seventh intercostal space, yielding a total of 2400 mL of hemorrhagic pleural fluid. The drainage was maintained under gravity (siphon) drainage.

**Table 1:** Bood and pleural biological parameters Values in bold are above the reference range.

Biological parameter	Value	Reference values
Pleural hematocrit (PH)	29.5%	≈0% (≪1%)
Serum hematocrit (SH)	34.3%	Male: 40-50%
Ratio PH/SH	86%	< 1% (hémothorax if > 50%)
Serum tumor marker CA 125	49.5 U/mL	<35 U/mL
Serum tumor marker CA 15-3	68.7 U/mL	<30 U/mL
Pleural Hyaluronic acid	12,950 µg/L	<10,000 µg/L

**Figure 2:**
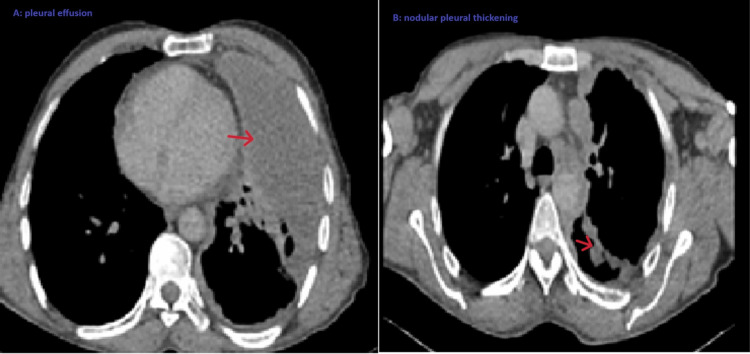
A chest CT scan with mediastinal window revealed nodular pleural thickening on the left side, associated with a moderate left pleural effusion and mediastinal lymphadenopathy. A: The red arrow shows the pleural effusion. B: The red arrow shows the nodular pleural thickening.

**Figure 3:**
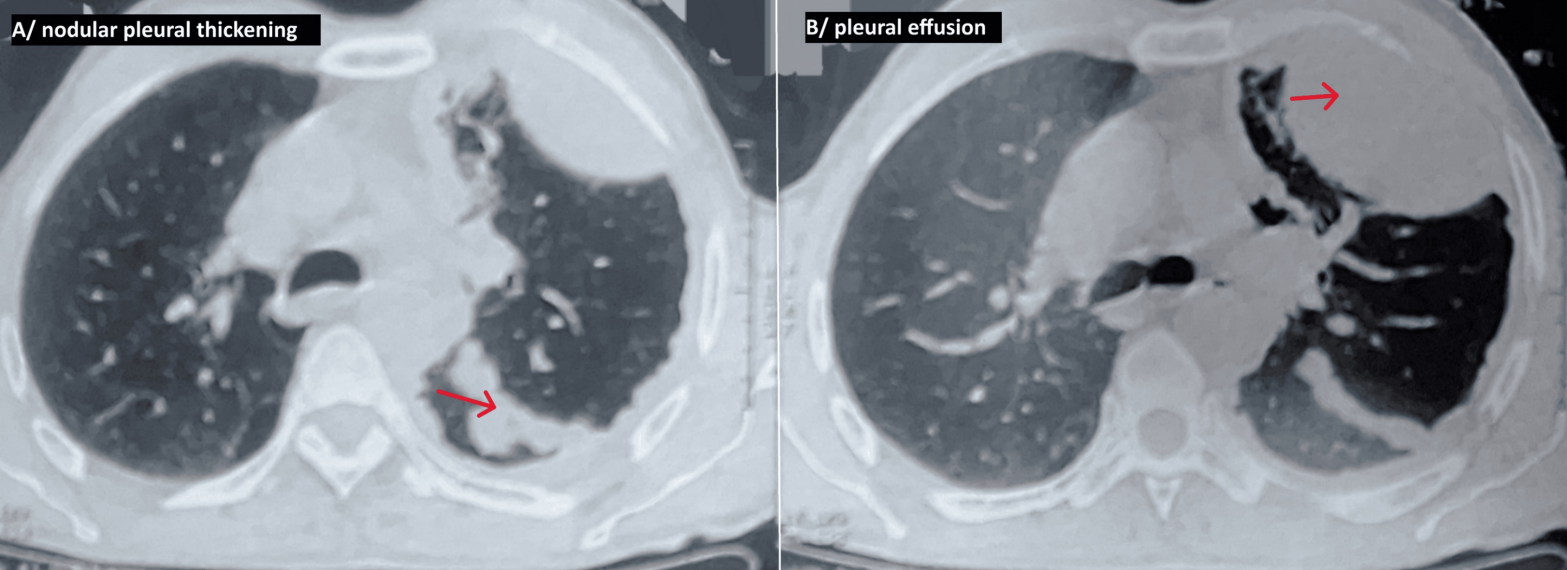
A chest CT scan with parenchymal window revealed nodular pleural thickening on the left side, with a moderate left pleural effusion, and emphysematous lungs. A: The red arrow marks the nodular pleural thickening. B: The red arrow marks the moderate pleural effusion.

A chest CT scan (Figures 2,3) revealed a nodular pleural thickening up to 19.5 mm, large loculated left subpleural collection (19.8 cm), mediastinal lymphadenopathy, emphysematous changes, and no soft tissue or bone invasion.

Abdominopelvic CT scan with no evidence of secondary abdominal lesions. Bone scintigraphy showed a single secondary osseous lesion involving the D2 vertebral body.

Bronchoscopy showed mild, diffuse bilateral bronchial inflammation with no endobronchial mass or granuloma. Bronchial aspirate was negative for tuberculosis, and cytology revealed no malignant cells. Routine laboratory tests were normal except for elevated LDH. PSA was normal, while tumor markers CA 125 and CA 15-3 were slightly elevated (Table 1).

Biochemical analysis of the pleural fluid showed a high protein level, a hemorrhagic appearance, sterile culture, and negative results for *Mycobacterium tuberculosis* and GeneXpert. Hyaluronic acid was markedly elevated (Table 1).

**Figure 4:**
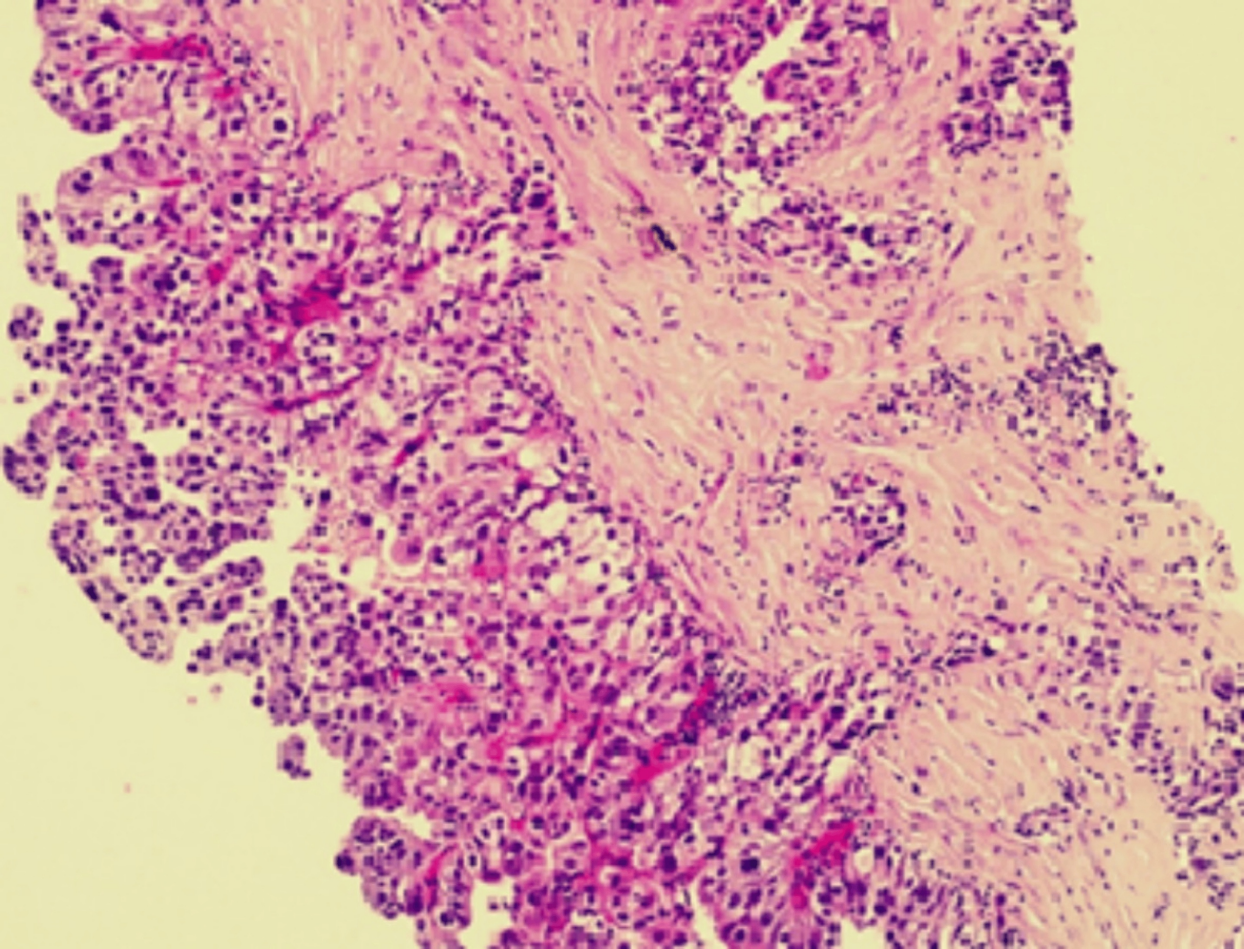
The histological study of the pleural biopsy.

Cytological examination of the pleural fluid and histopathological analysis of the pleural biopsy revealed an atypical mesothelial-like proliferation (Figure 4).

Immunohistochemical findings confirmed pleural involvement by a pulmonary adenocarcinoma of the pseudomesotheliomatous subtype. Mesothelial markers (calretinin, CK5/6, WT1, GATA3) were negative, while epithelial markers showed strong positivity for TTF1, moderate positivity for Napsin A, and positivity for pan-cytokeratin (AE1/AE3) with a CK7+/CK20− profile.

Tissues were stained with hematoxylin and eosin. The histopathological examination presented glandular and cord-like structures of tumor cells within desmoplastic stroma. A hobnail-like appearance of tumor cells with hyperchromatic nuclei and inconspicuous nucleoli was identified.

**Figure 5:**
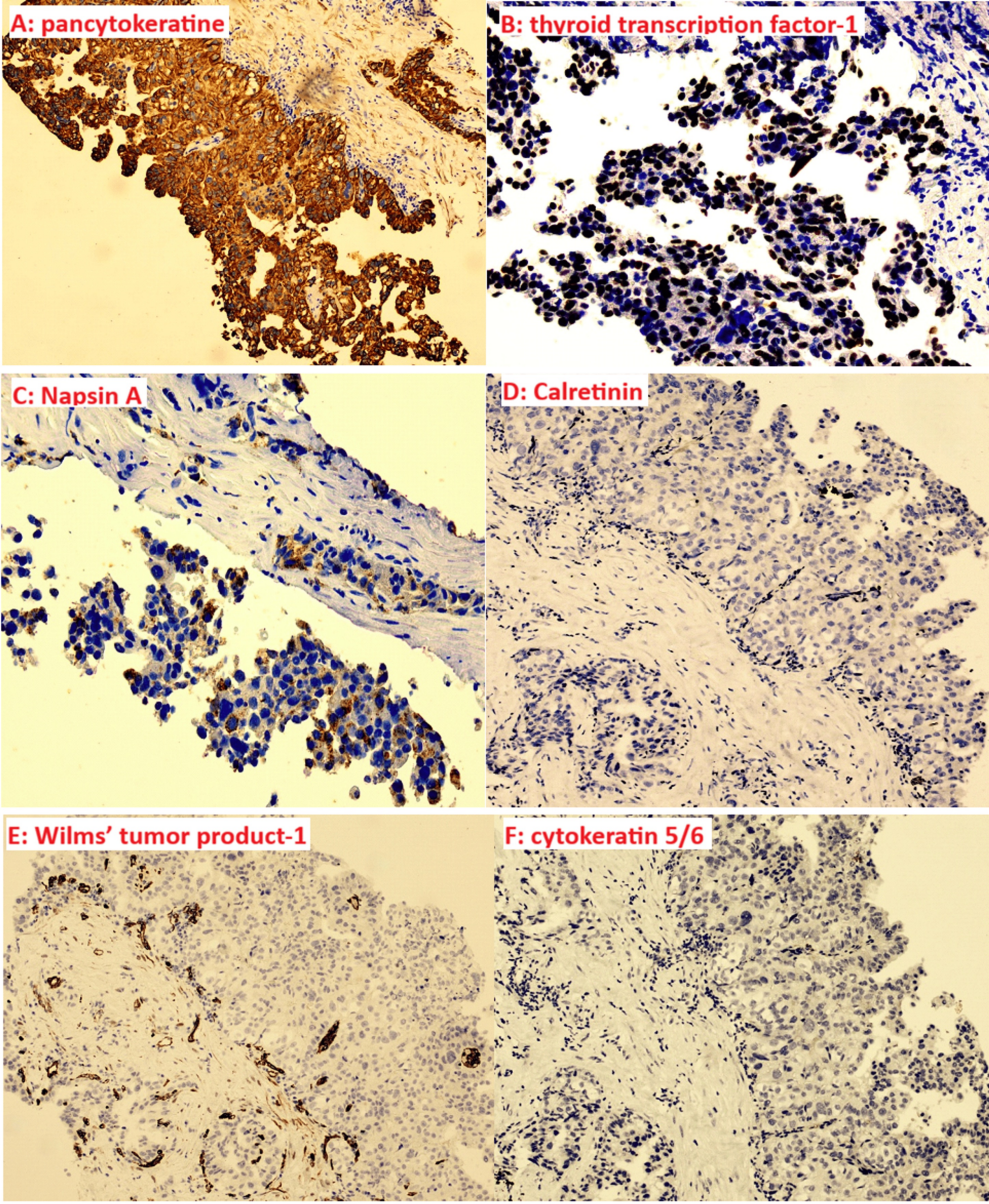
Immunohistochemical analysis of the pleural biopsy. The tumor cells were positive for pancytokeratine (A), and epithelial markers : thyroid transcription factor-1 (B) and Napsin A (C), but no reactivity for mesothelial markers calretinin (D), Wilms’ tumor product-1 (E), and cytokeratin 5/6 (F).

Immunohistochemically (Figure 5), the tumor cells were positive for pancytokeratine (Figure 5A), and epithelial markers: thyroid transcription factor-1 (Figure 5B) and Napsin A (Figure 5C), but no reactivity for mesothelial markers calretinin (Figure 5D), Wilms’ tumor product-1 (Figure 5E), and cytokeratin 5/6 (Figure 5F).

The final diagnosis could be made based mainly on immunocytochemical results. With these immunohistochemical results and clinical presentation, the tumor was diagnosed as pseudomesotheliomatous lung carcinoma.

As part of the pre-therapeutic workup, transthoracic echocardiography was normal. Spirometry showed no obstructive ventilatory defect, with a forced expiratory volume in one second (FEV1) of 1.75 L (55% of predicted).

Regarding oncogenic drivers, no mutations were found in epidermal growth factor receptor (EGFR), anaplastic lymphoma kinase (ALK), ROS proto-oncogene 1 (ROS1), rearranged during transfection (RET), mesenchymal-epithelial transition factor (MET), neurotrophic tyrosine receptor kinase (NTRK), B-Raf proto-oncogene (BRAF), or Kirsten rat sarcoma viral oncogene homolog (KRAS). Programmed death-ligand 1 (PD-L1) expression was negative, with a Tumor Proportion Score (TPS) of 0%. Given the absence of molecular alterations and a PD-L1 expression below 50%, in a 53-year-old patient with a WHO performance status of 0, it was deemed appropriate to initiate a combination chemotherapy regimen with cisplatin and vinorelbine. The patient died suddenly after two cycles of chemotherapy, in less than three weeks.

## Discussion

PCL was first described by Harwood et al. in 1976 [2] as a malignant tumor mimicking diffuse malignant pleural mesothelioma in radiological and histological features. It mainly affects men in their 60s and 70s, with many patients being smokers, Although traces of asbestos have been found in some cases and occupational exposure reported in about 21% of patients, the link between asbestos and PCL remains unclear, Because radiology cannot reliably distinguish PCL from mesothelioma, diagnosis requires histopathology and immunohistochemistry, PCL tumor cells express markers such as TTF-1, Napsin A, epithelial membrane antigen (EMA), and carcinoembryonic antigen (CEA), but are negative for mesothelial markers like calretinin and D2-40 [7], In our case, the patient was a chronic smoker, 53 years old (relatively younger than the typical age) and had no known exposure to asbestos. The diagnosis was confirmed by immunohistochemical analysis, which showed negative mesothelial markers.

Hemothorax is an uncommon clinical manifestation of PCL, which more commonly presents with pleural effusion and diffuse pleural thickening [8]. In our case, the presentation was a hemothorax, with pleural and fissural thickening observed.

Snaebjornsson et al. reported a rare case of PLC presenting as a massive hemothorax in a 74-year-old patient without asbestos exposure, with progressive shortness of breath and a large right-sided pleural effusion but no visible lung mass. Tumor invasion of pleural blood vessels caused uncontrollable bleeding, leading to death despite emergency surgery, highlighting this rare, life-threatening complication and the need for rapid multidisciplinary management [8]. In comparison with our patient, the thoracic drainage was able to control the hemothorax, removing a total of 2400 ml of hemorrhagic fluid, with clinical improvement.

An et al. reported the case of a 69-year-old male smoker presenting with dyspnea and recurrent pleural effusions, who was diagnosed by biopsy and immunohistochemistry with PCL. Imaging showed pleural thickening without a pulmonary mass. The patient refused chemotherapy and is receiving symptomatic treatment [1]. In our patient’s case, he was treated with chemotherapy based on Cisplatin and Vinorelbine.

Shah et al. reported three cases of poorly differentiated, pleura-based tumors in older white male patients presenting with chest pain, weight loss, and extensive pleural involvement, with some having a smoking history or asbestos exposure. Immunohistochemical analysis, particularly using Ber-EP4 and B72.3, was crucial in distinguishing these tumors from mesothelioma, a finding supported by a review of 65 similar cases [9]. In our patient, the initial presentation with nodular pleural thickening, mesothelial proliferation on histological examination, and elevated hyaluronic acid levels was suggestive of mesothelioma. However, immunohistochemistry completely shifted the diagnosis.

Saleh et al. reported six cases of primary lung cancers that closely mimicked malignant pleural mesothelioma. Clinical, radiologic, and gross examinations were insufficient for differentiation. Routine histology was often inconclusive. Diastase-periodic acid-Schiff (DPAS) staining proved crucial, showing mucin positivity in all lung cancers except one, which was absent in mesotheliomas. All tumors were glandular, mostly bronchiolar carcinomas, with some small subpleural tumors spreading via lymphatics. The study highlights the need for specialized histochemical techniques to accurately distinguish pseudomesotheliomatous carcinoma from mesothelioma [5]. In our patient, histopathology revealed glandular and cord-like tumor structures within desmoplastic stroma, with characteristic hobnail-like tumor cells featuring hyperchromatic nuclei and small nucleoli.

Vuković et al. reported a case of a 64-year-old former smoker with cough, chest pain, fatigue, dyspnea, sweating, and high fever. He was initially treated with probabilistic antibiotic therapy without specifying the molecule and the treatment failed. Imaging showed pleural thickening, large effusion, and subpleural consolidation. Pleural fluid cytology revealed malignant cells, but the distinction between metastatic adenocarcinoma and mesothelioma was unclear. Pleural biopsy with histochemical and immunohistochemical analysis confirmed pseudomesotheliomatous lung adenocarcinoma [10].

Treatment of pseudomesotheliomatous tumors is mainly based on chemotherapy, sometimes combined with palliative radiotherapy or pleurodesis for effusion control. The prognosis is poor, with an average survival of six to eight months, similar to malignant mesothelioma (nine months in Katgi et al.’s case) [11]. Our patient survived approximately one month after diagnosis while receiving chemotherapy treatment.

Pardo et al. emphasize the importance of raising awareness of this rare entity among pathologists to avoid misdiagnosis. They reported that despite complete surgery and chemotherapy in two cases, all patients died within 14 months [12].

## Conclusions

The clinical and radiological presentation of pseudomesotheliomatous tumors remains misleading. It is emphasized that the absence of a visible pulmonary mass does not confirm a primary pleural origin; a diagnosis of mesothelioma cannot be made without formal histological evidence. In the presence of diffuse pleural thickening, lung carcinomas with pleural tropism must also be considered. Radiological, clinical, and histological correlation is essential, with immunohistochemistry playing a crucial role. The prognosis remains poor.

Increasing awareness and understanding of this entity will help improve patient management and guide future research toward more effective therapies.
